# Association between Vitamin D Receptor Gene Polymorphisms and Breast Cancer Risk: A Meta-Analysis of 39 Studies

**DOI:** 10.1371/journal.pone.0096125

**Published:** 2014-04-25

**Authors:** Kai Zhang, Lihua Song

**Affiliations:** 1 Department of Internal Medicine Oncology, Shandong Cancer Hospital and Institute, Shandong Academy of Medical Sciences, School of Medicine and Life Sciences, University of Jinan -Shandong Academy of Medical Sciences, Jinan, China; 2 Department of Internal Medicine Oncology, Shandong Cancer Hospital and Institute, Jinan, China; Ohio State University Medical Center, United States of America

## Abstract

**Background:**

The associations between vitamin D receptor (VDR) gene polymorphisms and breast cancer risk were comprehensively investigated to clarify issues that remain controversial.

**Methodology/Principal Findings:**

An electronic search was conducted of several databases, including PubMed, the Cochrane library, Web of Science, EMBASE, CBM and CNKI, for papers that describe the association between *Fok1,* poly-A repeat, *Bsm1, Taq1* or *Apa1* polymorphisms of the *VDR* gene and breast cancer risk. Summary odds ratios and 95% confidence intervals (CI) were estimated based on a fixed-effect model (FEM) or random-effect model (REM), depending on the absence or presence of significant heterogeneity. A total of 39 studies met the inclusion criteria. A meta-analysis of high-quality studies showed that the *Fok1* polymorphism of the *VDR* gene was associated with an increased risk of breast cancer (*ff* vs. *Ff*+*FF*, OR: 1.09, 95%CI: 1.02 to 1.16, p = 0.007). No significant associations were observed between the other polymorphisms and breast cancer risk. No positive results were detected by pooling the results of all relevant studies.

**Conclusion:**

A meta-analysis of high-quality studies demonstrated that the *Fok1* polymorphism of the *VDR* gene was closely associated with breast cancer risk.

## Introduction

Laboratories investigations and epidemiological studies have suggested that the level of vitamin D, and the expression of the vitamin D receptor (VDR), might be associated with an increased risk of breast cancer [1], [2]. However, based on the current available data, these relationships need to be further evaluated. Vitamin D from all sources undergoes hydroxylation in the liver to become 25-hydroxyvitamin D [25(OH) D], which is then further hydroxylated in the kidneys and other tissues to an active form of vitamin D (1,25-dihydroxyvitamin D, 1,25(OH)_2_D) [3], [4]. In several studies, 1,25(OH)_2_D has been demonstrated to promote cell differentiation and inhibit cell proliferation, potentially modifying cancer risk via binding to the VDR [5], [6]. The VDR is an intracellular hormone receptor that specifically binds to 1,25(OH)_2_D and interacts with specific nucleotide sequences (response elements) of target genes to produce a variety of biological effects. As vitamin D exerts its activity by binding to the VDR, the finding that normal breast epithelial cells [7] and most breast cancer cells [8] express VDR suggests the possibility that VDR gene polymorphism may be associated with breast cancer risk.

The gene that encodes VDR maps to the long arm of chromosome 12 (12q12-14), and harbors approximately 200 single nucleotide polymorphisms (SNPs). Some are linked to differences in 1-25(OH)_2_D uptake and can therefore be considered as latent disease risk variants. A series of characterized *VDR* gene polymorphisms, including *Fok1* (rs2228570) [9]–[24], a poly-adenosine (poly-A) repeat variant [10], [19], [22], [25]–[27], *Bsm1* (rs1544410) [10], [11], [13], [15]–[17], [19]–[22], [24], [25], [28]–[33], *Taq1* (rs731236) [9], [12]–[14], [17], [18], [24], [26], [29], [30], [34]–[38] and *Apa1* (rs7975232) [9], [13], [17], [18], [24], [29], [36], [38]–[40], have been extensively studied with regard to their association with breast cancer risk, but with conflicting results. To clarify the association between breast cancer risk and *VDR* gene polymorphisms, we performed a meta-analysis of 39 existing studies to clarify the relationship between genetic variations in *VDR* and the risk of breast cancer.

## Materials and Methods

### Search Strategies

A comprehensive literature search of numerous databases, including PubMed, the Cochrane library, Web of Science, EMBASE, CBM (China Biology Medicine) and CNKI (China National Knowledge Infrastructure), was conducted up until December 21^st^, 2013. Publications with the following search words in the titles, abstract or key words of the original studies were included: ‘vitamin D receptor’, ‘VDR’, ‘*Fok1*’, ‘Poly A’, ‘*Bsm1*’, ‘*Taq1*’, ‘*Apa1*’, ‘polymorphism’ or ‘variant’ or ‘mutation’ coupled with the term ‘breast cancer’. Additional studies that were not captured by the database search were identified by reviewing the bibliographies of relevant articles.

### Inclusion Criteria

All identified studies were reviewed independently by two investigators. The following criteria were used for a publication to be included in the meta-analysis: (1) any study published as an original study that evaluated the association between *VDR* gene polymorphisms (*Fok*1, poly A, *Bsm*1, *Taq*1 and *Apa*1) and breast cancer risk; (2) cases of breast cancer were confirmed by medical records or linkage with population-based tumor registries; (3) the numbers of case and control groups for each genotype were reported or the relevant data was available, and adequate data was provided to calculate the odds ratio (OR); and (4) publications in both English and Chinese were included.

### Data Extraction and Quality Assessment

Two investigators conducted the search, extracted and tabulated all the relevant data independently. If a study was referenced more than once, the most complete and newly released study was used. If one article reported two or more different case-control studies, it was considered as two or more studies, respectively. Data extracted from each study were as followings: name of the first author, publication year, ethnic origin of the studied population, numbers of case and controls, and the genotype frequency of the polymorphisms. To maintain consistency with the previously published literature, SNPs of the *VDR* gene were reported using restriction fragment length polymorphism (RFLP) nomenclature for the major and minor alleles, as follows: *Fok1* (rs2228570) alleles C = F and T = f; *Bsm1* (rs1544410) alleles G = b and A = B; *Taq1* (rs731236) alleles T = T, and C = t; and *Apa1* (rs7975232) A = A and C = a. The allele counts were calculated from the genotype counts when needed.

The quality of studies was assessed according to the STrengthening the REporting of Genetic Association Studies (STREGA) criteria [41], and studies according with STREGA criteria were defined as high-quality studies. An independent review and decision was made by a senior investigator if there were disagreements between the two initial reviewers.

### Statistical Analysis

The strengths of the associations between five polymorphisms of the *VDR* gene and the risk of breast cancer were assessed for the contrast between two groups of homozygotes (*ff* vs. *FF*, *SS* vs. *LL*, *bb* vs. *BB*, *tt* vs. *TT*, *aa* vs. *AA*), the recessive (*ff* vs. *Ff*+*FF*, SS vs. SL+LL, *bb* vs. *Bb*+*BB*, *tt* vs. *Tt*+*TT*, *aa* vs. *Aa*+*AA*), dominant (*ff*+*Ff* vs. *FF*, *SS*+*SL* vs. *LL*, *bb*+*Bb* vs. *BB*, *tt*+*Tt* vs. *TT*, *aa*+*Aa* vs. *AA*) and allelic (*f* vs. *F*, *S* vs *L*, *b* vs. *B*, *t* vs. *T*, *a* vs. *A*) models by calculating the pooled OR and its 95% confidence interval (CI). The pooled ORs were obtained using either the fixed-effects (Mantel-Haenszel’ method) model [42] or the random-effect (DerSimonian and Laird method) model [43], depending on the absence or presence of significant heterogeneity. The significance of pooled ORs was determined by the Z test. Heterogeneity among studies was assessed by the Chi-square test -based *Q* statistic and was quantified using the *I^2^* statistic [44]. A significant Q statistic (P-value <0.10) or I^2^ statistic (I^2^>50%) indicated significant heterogeneity existed across studies.

A Sensitivity analysis was performed to evaluate the key studies that had substantial impacts on between-study heterogeneity levels by removing the individual studies sequentially. To further explore the cause of heterogeneity, a meta-regression was performed, which included covariates such as ethnicity and sample size of the studies. If the origin of heterogeneity was found, subgroup analyses were conducted according to the origin. All statistical analyses, except the meta-regression, were performed using RevMan version 5.1.6 software (Review Manager, Copenhagen: the Nordic Cochrane Centre, The Cochrane Collaboration, 2011). The meta-regression procedure was conducted using STATA statistical software (version 12.0; Stata Corporation, College Station, USA).

The possibility of publication bias was assessed using Begger’s linear regression and funnel plots. An asymmetrical funnel plot suggested a possible publication bias.

## Results

### Baseline Characteristics

**Figure 1:**
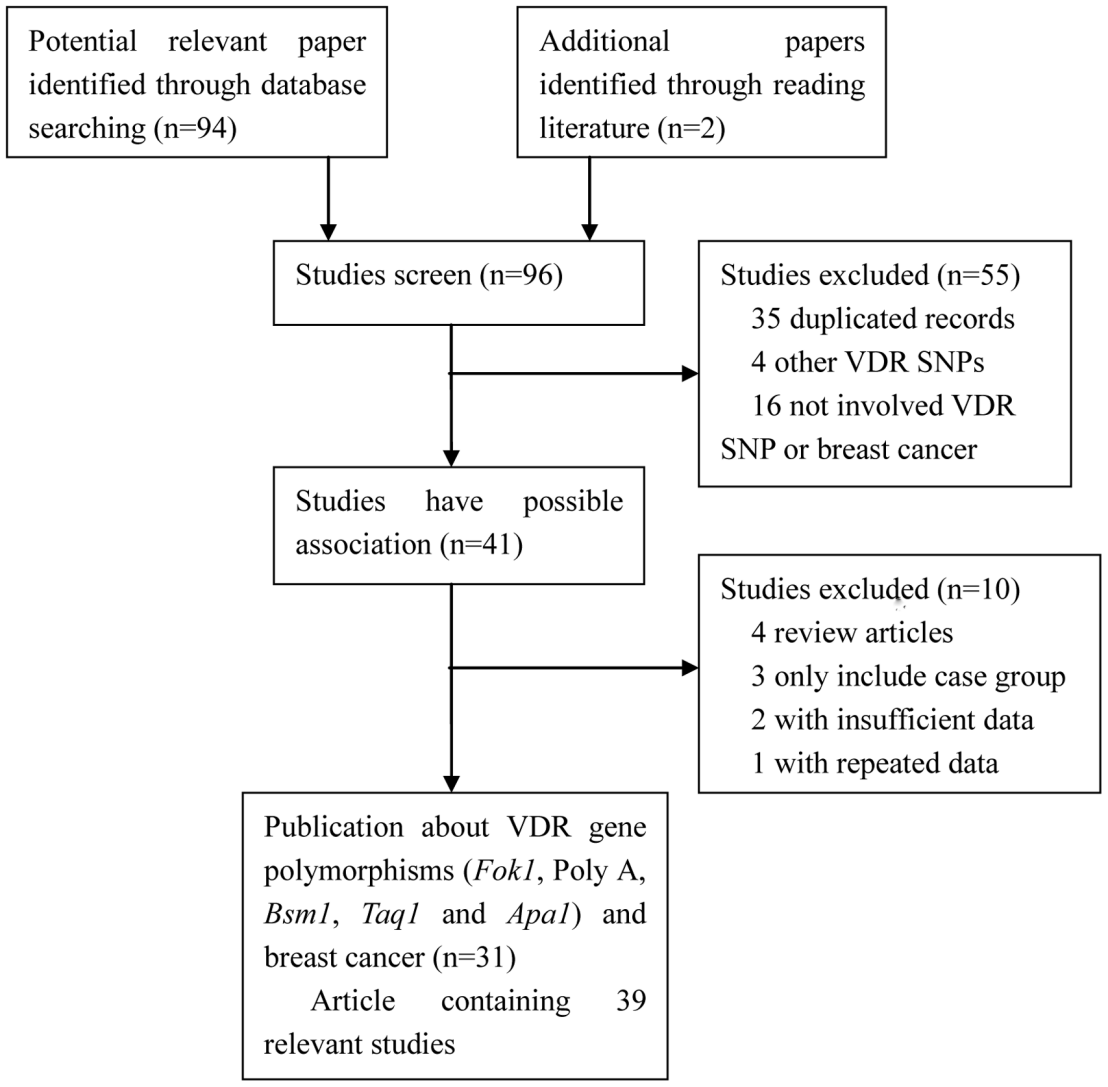
Flow diagram of the literature search.

A flow chart of the literature search is shown in Figure 1. According to the criteria eligibility, 39 studies was identified regarding the associations between the *Fok1,* poly-A, *Bsm1*, *Taq1* or *Apa1* polymorphisms of *VDR* gene and breast cancer risk. Among these studies, 22 studies [9]–[24] concerned the association of the *Fok1* polymorphism with breast cancer, including 16,353 cases and 21,881 controls, while seven studies [10], [19], [22], [25]–[27] investigated the association between the poly-A repeat variation and breast cancer risk, with 5,493 cases and 5,566 controls. For the *Bsm1* polymorphism, 25 studies [10], [11], [13], [15]–[17], [19]–[22], [24], [25], [28]–[33] included 16,160 cases and 21,023 controls, while 16 studies [9], [12]–[14], [17], [18], [24], [26], [29], [30], [34]–[38] on the *Taq1* polymorphism included 6,940 cases and 8,267 controls. For the *Apa1* polymorphism, 11 studies were included [9], [13], [17], [18], [26], [29], [36], [39] with 3,738 cases and 4,489 controls. All of these 39 studies provided sufficient data to calculate the possible relationship between the five polymorphisms of the *VDR* gene and breast cancer risk. The general characteristics of the selected studies are summarized in Table 1. More detailed information is shown in Tables S1, S2, S3, S4, S5. The pooled results are shown in Table 2.

**Table 1:** Characteristics of studies included in the meta-analysis of the relation between the Fok1,Poly A, Bsm1,Taq1 and Apa1 polymorphisms in the vitamin D receptor gene and breast cancer.

Author[Ref]	Year	Country	Racialdescent	Breast cancer	Control	Genotyping method	SNPs
Ruggiero et al. [28]	1998	Italy	European	88	167	PCR-RFLP	Bsm1
Curran et al. [9]	1999	Australia	European	135	110	PCR-RFLP	Fok1,Taq1, Apa1
Dunning et al. [34]	1999	UK	European	211	268	PCR-RFLP	Taq1
Dunning et al. [34]	1999	UK	European	740	359	PCR-RFLP	Taq1
Lundin et al. [35]	1999	Sweden	European	111	130	PCR-RFLP	Taq1
Ingel et al. [10]	2000	America	European	143	300	TaqMan	Fok1, poly A, Bsm1
Cui et al. [38]	2001	China	Asian	86	134	PCR-RFLP	Taq1, Apa1
Hou et al. [29]	2002	Taiwan	Asian	34	169	PCR-RFLP	Bsm1, Taq1, Apa1
Buyru et al. [30]	2003	Turkey	European	78	27	PCR-RFLP	Taq1
Guy et al. [22]	2004	UK	European	398	427	PCR-RFLP	Fok1, poly A
Hefler et al. [31]	2004	Germany	European	290	1699	PCR-RFLP	Bsm1
Sillanpaa et al. [36]	2004	Finnish	European	472	479	PCR-RFLP	Taq1, Apa1
Chen et al. [11]	2005	Tukey	European	1234	1676	TaqMan	Fok1, Bsm1
Lowe et al. [32]	2005	UK	European	179	179	PCR-RFLP	Bsm1
Vandevord et al. [33]	2006	America	Mixed	220	192	PCR-RFLP	Bsm1
John et al. [12]	2007	America	Mixed	764	865	PCR-RFLP	Fok1, Taq1,
McCullough et al. [13]	2007	America	European	475	480	TaqMan	Fok1, Bsm1, Taq1, Apa1
Trabert et al. [25]	2007	America	European	1139	905	PCR-RFLP	poly A, Bsm1
Trabert et al. [25]	2007	America	European	441	417	PCR-RFLP	poly A, Bsm1
Wedren et al [27]	2007	Sweden	European	1801	1712	TaqMan	poly A
Abbas et al. [14]	2008	Germany	European	1408	2612	PCR-RFLP	Fok1, Taq1,
Sinotte et al. [15]	2008	Canada	European	255	463	TaqMan	Fok1, Bsm1
Sinotte et al. [15]	2008	Canada	European	622	974	TaqMan	Fok1, Bsm1
Chakraborty et al. [26]	2009	India	Asian	160	140	PCR-RFLP	poly A, Taq1, Apa1
Mckay et al. [16]	2009	Unknown	European	1677	2795	TaqMan	Fok1, Bsm1
Mckay et al. [16]	2009	Unknown	European	1598	1952	TaqMan	Fok1, Bsm1
Mckay et al. [16]	2009	America	European	1073	1108	TaqMan	Fok1, Bsm1
Mckay et al. [16]	2009	America	European	685	683	TaqMan	Fok1, Bsm1
Mckay et al. [16]	2009	America	European	499	504	TaqMan	Fok1, Bsm1
Mckay et al. [16]	2009	America	European	1257	1748	TaqMan	Fok1, Bsm1
Li et al. [23]	2010	China	Asian	81	78	PCR-RFLP	Fok1
Anderson et al. [17]	2011	Canada	European	1560	1633	PCR-RFLP	Fok1, Bsm1, Taq1, Apa1
Dalessandri et al. [36]	2012	Canada	European	164	174	PCR-RFLP	Apa1
Liu et al. [37]	2011	China	Asian	80	80	PCR-RFLP	Taq1
Engel et al. [18]	2012	America	European	293	586	PCR-RFLP	Fok1, Taq1, Apa1
Huang et al. [40]	2012	China	Asian	146	320	TaqMan	Apa1
Rollison et al. [19]	2012	America	European	1740	2051	PCR-RFLP	Fok1,PolyA, Bsm1
Fuhrman et al. [21]	2013	America	European	477	842	TaqMan	Fok1, Bsm1
Mirash et al [24]	2013	Amierica	Mixed	232	349	PCR-RFLP	Fok1, Bsm1, Taq1, Apa1
Shahabazi et al. [20]	2013	Iran	Asian	140	156	PCR-RFLP	Fok1, Bsm1

PCR-RFLP: Polymerase chain restriction fragment length polymorphism.

**Table 2:** The pooled measures on the relation of Fok1, Poly A, Bsm1, Taq1 and Apa1 polymorphisms with breast cancer.

VDR polymorphism	Studies	Comparisons	Numbers of cases/controls	Pooled OR (95% CI)	P	I^2^	P_h_
Fok1	ALL relevant studies	*ff* vs. *FF*	16353/21881	1.06 (0.95–1.17)	0.30	57%	0.0005
	22	*ff*+*Ff* vs. *FF*	16353/21881	1.03 (0.97–1.09)	0.34	35%	0.06
		*ff* vs. *Ff*+*FF*	16353/21881	1.04(0.96–1.14)	0.34	50%	0.004
		*f* vs. *F*	16353/21881	1.03 (0.98–1.08)	0.29	54%	0.001
	Studies with high-quality	*ff* vs. *FF*	14076/19267	1.10 (1.00–1.21)	0.06	45%	0.03
	16	*ff*+*Ff* vs. *FF*	14076/19267	1.03 (0.97–1.10)	0.33	44%	0.03
		*ff* vs. *Ff*+*FF*	14076/19267	**1.09 (1.02–1.16)**	0.007*	18%	0.29
		*f* vs. *F*	14076/19267	1.04 (0.99–1.09)	0.12	52%	0.009
Poly-A	ALL relevant studies	*SS* vs. *LL*	5493/5566	0.99 (0.77–1.29)	0.96	74%	0.0009
	7	*SS*+*SL* vs. *LL*	5493/5566	0.99 (0.83–1.20)	0.96	76%	0.0003
		*SS* vs. *SL*+*LL*	5493/5566	1.04 (0.88–1.27)	0.66	49%	0.07
		*S* vs. *L*	5493/5566	1.00 (0.85–1.18)	0.98	77%	0.0005
	Studies with high-quality	*SS* vs *LL*	3474/3089	0.94 (0.71–1.25)	0.69	67%	0.03
	4	*SS*+*SL* vs. *LL*	3474/3089	0.95 (0.80–1.14)	0.60	64%	0.04
		*SS* vs. *SL*+*LL*	3474/3089	0.98 (0.78–1.23)	0.84	59%	0.006
		*S* vs. *L*	3474/3089	0.97(0.84–1.12)	0.67	71%	0.02
Bsm1	All relevant studies	*bb* vs. *BB*	16160/21203	1.07 (0.97–1.17)	0.18	44%	0.01
	25	*bb*+*Bb* vs. *BB*	16160/21203	1.03 (0.94–1.13)	0.49	54%	0.0007
		*bb* vs. *Bb* +*BB*	16160/21203	1.05 (0.97–1.14)	0.21	66%	<0.00001
		*b* vs. *B*	16160/21203	1.04 (0.98–1.09)	0.18	56%	0.003
	Studies with high-quality	*bb* vs. *BB*	11594/14404	1.03(0.93–1.14)	0.53	41%	0.06
	12	*bb*+*Bb* vs. *BB*	11594/14404	1.03 (0.94–1.14)	0.50	49%	0.02
		*bb* vs. *Bb* +*BB*	11594/14404	1.00(0.93–1.07)	0.96	48%	0.03
		*b* vs. *B*	11594/14404	1.01(0.96–1.06)	0.70	48%	0.03
Taq1	All relevant studies	*tt* vs. *TT*	6940/8267	1.02 (0.92–1.13)	0.66	0%	0.52
	16	*tt*+*Tt* vs. *TT*	6940/8267	1.03 (0.92–1.15)	0.61	47%	0.02
		*tt* vs. *Tt*+*TT*	6940/8267	0.98(0.90–1.08)	0.94	49%	0.02
		*t* vs. *T*	6940/8267	1.00 (0.92–1.08)	0.94	49%	0.02
Apa1	All relevant studies	*aa* vs. *AA*	3738/4489	0.99(0.87–1.13)	0.89	15%	0.31
	11	*aa*+*Aa* vs. *AA*	3738/4489	0.98(0.82–1.17)	0.82	61%	0.004
		*aa* vs. *Aa*+*AA*	3738/4489	1.00 (0.90–1.22)	0.99	0%	0.56
		*a vs. A*	3738/4489	0.95 (0.82–1.10)	0.52	75%	<0.0001

HWE: Hardy Weinberg Equilibrium.

P_h:P_ for heterogeneity, heterogeneity was checked by the chi square based Q test.

The symbol *shows the positive result.

### Meta-analysis

#### *Fok1* polymorphism and breast cancer risk

**Figure 2:**
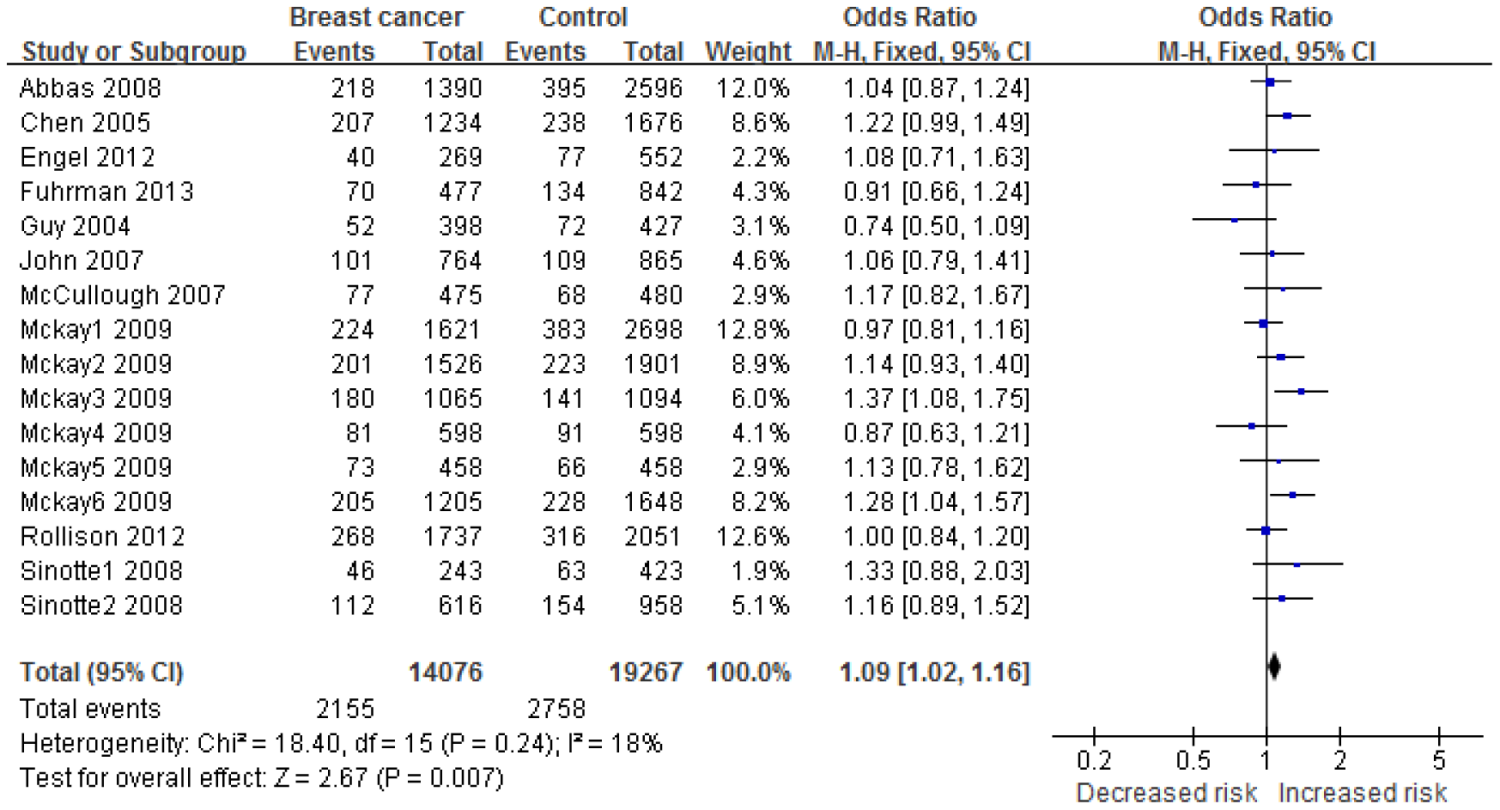
Forest plots of association of *Fok1* polymorphism with breast cancer. Significant association was detected between the genotype *ff* and breast cancer in recessive model (*ff* vs. *Ff*+*FF*). The squares and horizontal lines correspond to OR and 95% CI of specific study, and the area of squares reflects study weight. The diamond represents the pooled OR and 95% CI. Heterogeneity was checked by the chi square based Q test.

Sixteen of 22 studies were in accordance with STREGA criteria and were therefore defined as high-quality studies [11]–[16], [18], [19], [21], [22]. The meta-analysis of these studies showed a significant effect of the *ff* genotype on risk of breast cancer (*ff* vs. *Ff*+*FF* OR: 1.09, 95%CI: 1.02 to 1.16, p = 0.007; I^2^ = 18%, p_h_ = 0.24) (Figure 2). No significant associations were found for the other comparisons (*ff* vs. *FF* OR: 1.10, 95% CI: 1.00 to 1.20, p = 0.06; *ff*+*Ff* vs. *FF* OR: 1.03, 95% CI: 0.97 to 1.10, p = 0.33; *f* vs. *F* OR: 1.04, 95% CI: 0.99 to 1.09, p = 0.12 (Figures S1, S2, S3). No positive results were detected by pooling the data from all 22 studies.

#### Poly-A variant and breast cancer risk

Four of the seven studies complied with the STREGA criteria [22], [25], . No significant association was detected between the poly-A variant and breast cancer by pooling the results of all studies or only high-quality studies.

#### *Bsm1*, *Taq1* and *Apa1* polymorphism and breast cancer risk

For all relevant studies, the pooled results did not illustrate any significant correlation between *Bsm1*, *Taq1* or *Apa1* polymorphisms and breast cancer risk. Twelve high-quality studies did not show a significant association between the *Bsm1* polymorphism and breast cancer risk [11], [13], [15]–[17], [19], [21], [24]. As less than three studies complied with the STREGA criteria for the *Taq1* and *Apa1* polymorphisms, and therefore meta-analyses were not performed by pooling the results of high-quality studies alone.

### Meta-regression

To detect the origin of study heterogeneity, the random effects meta-regression method was used [45]. In the regression procedure, an independent variable, the logarithm OR, and two covariates, ethnicity and sample size was included. The results of all the meta-regressions showed that the two covariates were not the origin of the heterogeneity.

### Sensitivity Analysis and Publication Bias Evaluation

**Figure 3:**
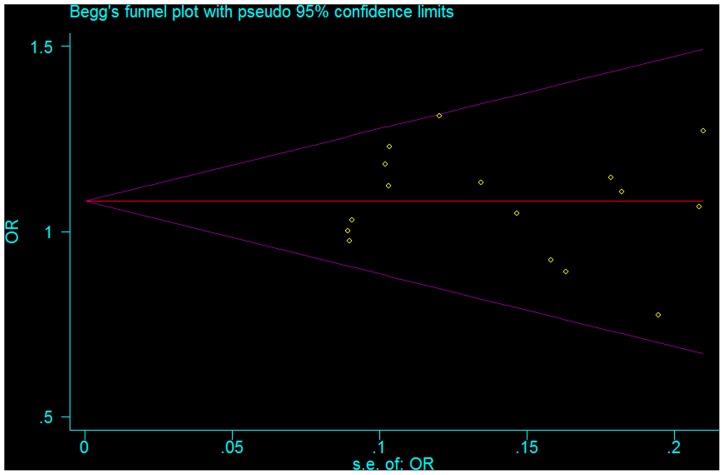
Begg’s funnel plot to examine publication bias for comparisons of *Fok1* polymorphism (*ff* vs. *Ff*+*FF*).

A sensitivity analysis was performed by removing the individual studies sequentially to assess the effect of individual studies. The results detected did not differ from the initial analysis. Begger’s linear regression showed that no publication bias existed in relationship to any variation (P>0.05). The funnel plot for the recessive model of *Fok1* polymorphism was symmetrical (Figure 3). The other funnel plots were not shown.

## Discussion

Vitamin D regulates a variety of independent biological processes including bone metabolism, the innate immune response, cell proliferation and cell differentiation [46], [47]. Several studies have suggested that adequate vitamin D levels may provide protection against chronic diseases, such as cancers, and could improve cancer prognosis [48]. The important roles that *VDR* polymorphisms play in the pathogenesis of breast cancer have been investigated across the world. Many studies have been carried out to investigate the relationship between *VDR* gene polymorphisms and the risk of breast cancer [9]–[39]. As a result of the limitations of sample sizes and the low statistical power of individual studies, research results have been conflicting and inconsistent. A previous meta-analysis involved only four SNPs (*Fok1*, *Bsm1*, *Taq1* and *Apa1*) and contained relevant studies that were published before October 2008 [49]. Another review published in 2009 summarized the association between *VDR* polymorphisms and breast cancer risk, but no definitive quantitative results were obtained [50]. Our current meta-analysis included almost all studies that had investigated the associations between *Fok1*, poly-A, *Bsm1*, *Taq1* and *Apa1* polymorphisms and breast cancer risk. This is the most comprehensive meta-analysis to data, to the best of our knowledge.

This meta-analysis included data from 39 relevant studies. The meta-analysis of high-quality studies showed that individuals with homozygous *ff* genotype were responsive to the increased risk of breast cancer compared to patients with *Ff* or *FF* genotypes. The overall data from all genetic models did not demonstrated that there was a significant association between the poly-A repeat, *Bsm1*, *Taq1* and *Apa1* polymorphisms and breast cancer risk. A sensitivity analysis was performed by removing the individual studies sequentially, and the overall genetic effects were consistent with those of the corresponding sensitivity analyses for the poly-A, *Bsm1*, *Taq1* and *Apa1* variants. These findings further indicated the robustness of the lack of association between these four polymorphisms and breast cancer risk.

No linkage disequilibrium was shown between the *Fok1* polymorphism and *Bsm1*, *Taq1* and *Apa1* polymorphisms [51]. Therefore, *Fok1* can be considered as an independent marker within the *VDR* gene. The effect of this SNP on breast cancer is plausible, given that the presence of the *f* allele in the 5′-promoter region of the *VDR* gene results in a protein that is three amino acids longer protein that the wild-type, and which is less transcriptionally active [52]. The presence of this polymorphism could therefore cause reduced effects of vitamin D. Although most previous studies on the association of *Fok1* polymorphism with breast cancer did not identify any evidence for a significant association, McKay et al. found a positive statistically significant association between *ff* genotype and the increased risk of breast cancer by pooling six studies (OR: 1.16, 95%CI: 1.04 to 1.28, p = 0.006) [16]. Similarly, a previous meta-analysis published in 2009 also showed a significant increased risk of breast cancer in *ff* genotype carriers (OR: 1.15, 95%CI: 1.03 to 1.26, p = 0.010) [49]. A meta-analysis of high-quality studies, provided strong evidence that the *ff* genotype was significantly associated with risk of breast cancer, in accordance with previous reports [49].

The poly-A repeat in the 3′-untranslated region of the *VDR* gene which is strongly linked with *Bsm1*, *Apa1* and *Taq1* has an important impact on *VDR* mRNA stability [52]. Several studies have indicated that *LL* genotype (long/long) confers susceptibility to breast cancer risk compared with the *SS* genotype [10],[22],[26]. Chakraborty et al. revealed that the *LL* genotype is significantly associated with high-grade breast cancer in northern Indians [(unadjusted OR (95% CI): 4.45(1.87, 10.63); adjusted OR (95% CI): 4.66 (1.88, 11.53)] [26]. However, this result conflicted with the report from Ingles et al., where breast cancer risk was found to increase with increasing numbers of *S* alleles [10]. Our finding did not show any significant association between the poly-A variation and breast cancer risk in any genetic model. These inconsistent results might result from differences of ethnicity, sample size, study design, amongst other factors.

The *Bsm1* polymorphism is located at the 3′ end of the *VDR* gene. It does not appear to change the nature of the translated VDR protein [53]. However, this polymorphism is linked in a haplotype with the variable-length poly A sequence within the 3′-untranslated region, which affects the *VDR* mRNA stability [54]. On the other hand, the *Bsm1*, *Taq1* and *Apa1* polymorphisms are all in the same linkage disequilibrium block. These polymorphisms have been widely investigated, but with differing results. Consistent with a previous meta-analysis, our finding showed no significant association of these three genetic variations with breast cancer risk. Several studies have been performed to examine the *VDR* haplotypes [9],[13],[14],[18],[35], but these results were also conflicting. McCullough et al. has investigated haplotypes that involved *Bsm1*(*B/b*), *Apa1*(*A/a*), *Taq1*(*T/t*) and a poly-A repeat(*S/L*), but they failed to find significant association between any haplotype and breast cancer risk [13]. However, in a Caucasian population the *baTL* has been reported to increase the risk of breast cancer [9],[35]. It is unclear whether chance or underlying differences in populations led to these inconsistencies. Due to the limited information available about these polymorphisms, we could not conduct an analysis for linkage disequilibrium and haplotypes.

A few studies have investigated the association of *VDR* polymorphisms with breast cancer survival, but their results were also inconsistence. An analysis conducted among 111 Swedish breast cancer patients younger than 37 years of age found a trend towards a higher survival rate, especially among those estrogen receptor-positive tamoxifen-treated patients that were homozygous for the rare *Taq1* allele [35]. However, Perna et al. reported that homozygous carriers of the rare *Taq1* homozygous genotype had a 2.8-fold increase in the probability of death from breast cancer compared to homozygous carriers with the common allele (OR: 2.8, 95%CI: 1.1–7.2) [55].

Our meta-analysis illustrates strong evidence for the association between a *VDR* gene polymorphism in *Fok1* and an increased risk of breast cancer. The obvious evidence of between-study heterogeneity in this meta-analysis should be discussed. Although a meta-regression procedure that included two covariates was performed, the origin of the heterogeneity among the studies was not found. The heterogeneity might have been due to other factors, such as diversity in the population characteristics (ethnicity, age, sun exposure and dietary vitamin D intake, etc.), genotyping methods and study design. Previous studies have shown that the ethnic (genetic) background, gene-gene or gene-environment interactions and life-style (sun exposure, dietary vitamin D intake and smoking) might play a major role in the increased risk of breast cancer in association with genetic variations. Our meta-analysis was based on estimates without adjusting the data for these factors, which is another potential limitation of this study.

In conclusion, this comprehensive meta-analysis of high-quality studies provides substantial evidence that the *Fok1* polymorphism in the *VDR* gene is significantly associated with an increased risk of developing breast cancer. Furthermore, individuals that were homozygous for the minor allele genotype of *Fok1* were more likely to develop breast cancer. No correlations were found between the poly-A variation, *Bsm1*, *Taq1* and *Apa1* polymorphisms in the *VDR* gene and the risk of breast cancer in this study.

## Supporting Information

Figure S1 **Forest plots of association of** ***Fok1*** **polymorphism with breast cancer (** ***ff*** **vs.** ***FF*** **).**
(TIF)

Figure S2 **Forest plots of association of** ***Fok1*** **polymorphism with breast cancer (** ***ff*** **+** ***Ff*** **vs.** ***FF*** **).**
(TIF)

Figure S3 **Forest plots of association of** ***Fok1*** **polymorphism with breast cancer (** ***f*** **vs.** ***F*** **).**
(TIF)

Table S1 **Characteristics of studies included in this meta-analysis between the** ***Fok1*** **polymorphism in the vitamin D receptor gene and breast cancer.**
(DOCX)

Table S2 **Characteristics of studies included in this meta-analysis between the poly-A polymorphism in the vitamin D receptor gene and breast cancer.**
(DOCX)

Table S3 **Characteristics of studies included in this meta-analysis between the** ***Bsm1*** **polymorphism in the vitamin D receptor gene and breast cancer.**
(DOCX)

Table S4 **Characteristics of studies included in this meta-analysis between the** ***Taq1*** **polymorphism in the vitamin D receptor gene and breast cancer.**
(DOCX)

Table S5 **Characteristics of studies included in this meta-analysis between the** ***Apa1*** **polymorphism in the vitamin D receptor gene and breast cancer.**
(DOCX)

Appendix S1 **PRISMA Checklist.**
(DOC)
