# Supplementary figures and images for: Association between Vitamin D Receptor Gene Polymorphisms and Breast Cancer Risk: A Meta-Analysis of 39 Studies
Source: PLoS One. 2014 Apr 25;9(4):e96125. doi: 10.1371/journal.pone.0096125 (PMC4000223; doi:10.1371/journal.pone.0096125)

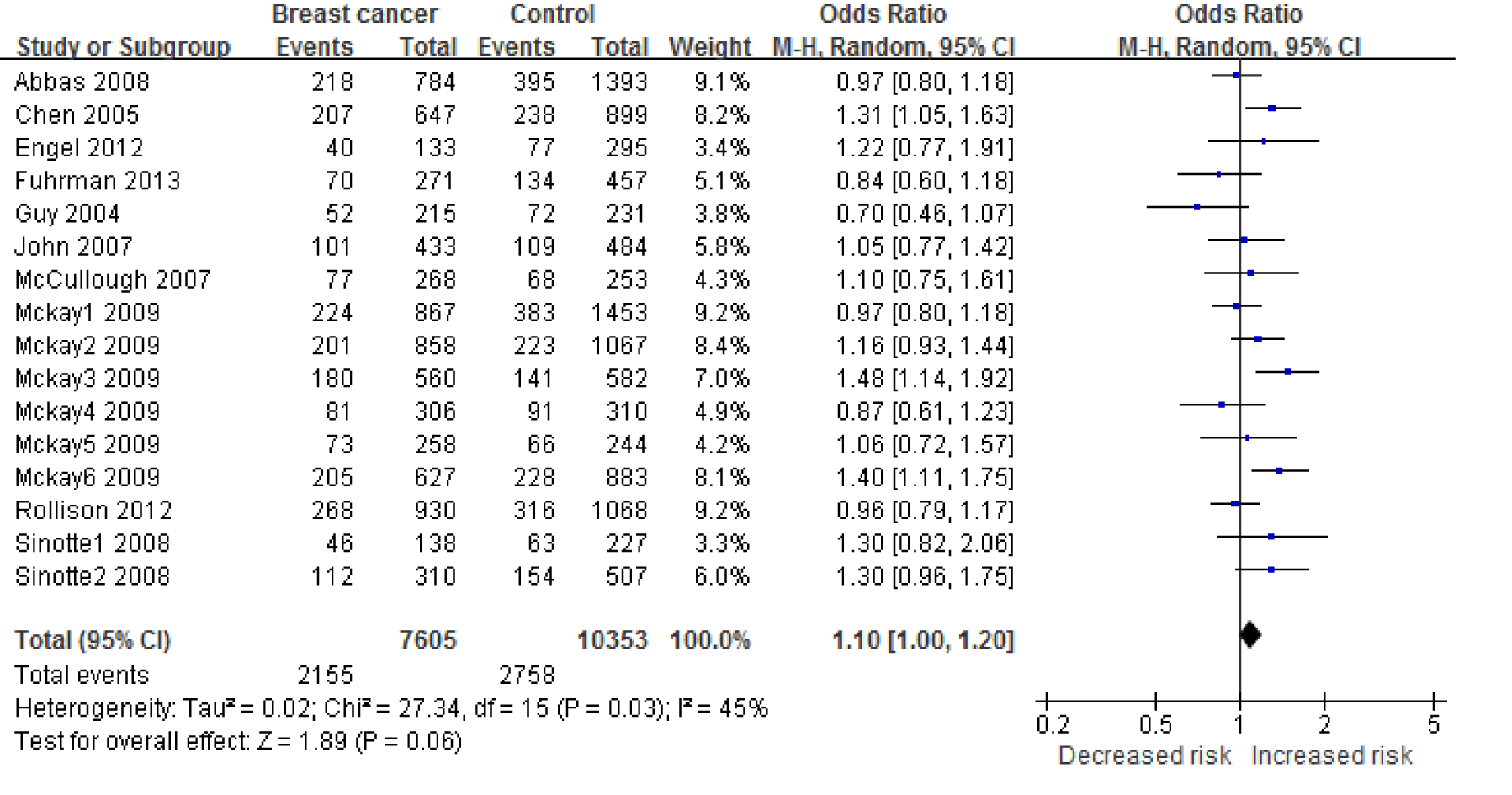

Supplement: Figure S1 — Forest plots of association of Fok1 polymorphism with breast cancer ( ff vs. FF ). (TIF) [file pone.0096125.s001.tif]

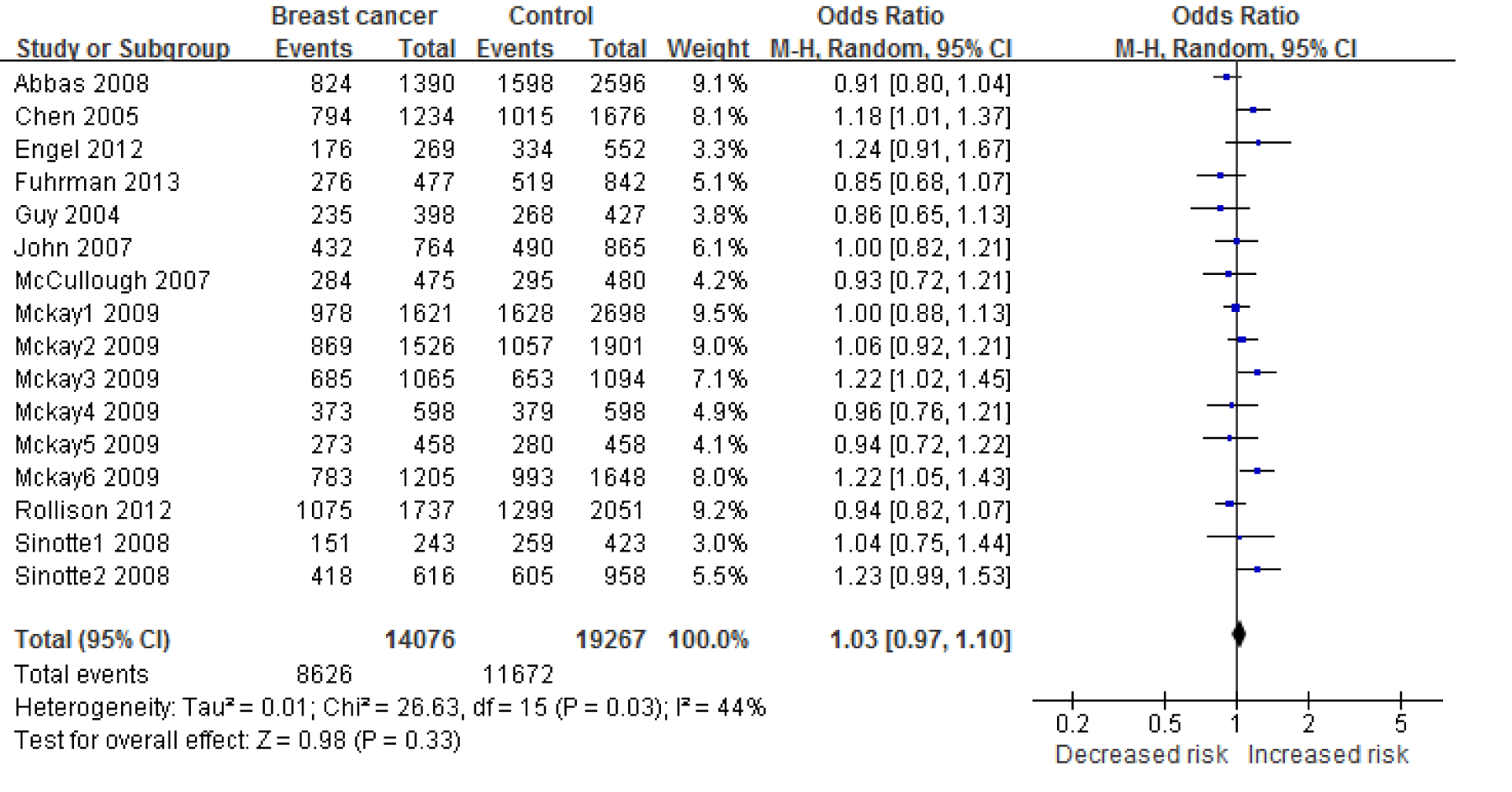

Supplement: Figure S2 — Forest plots of association of Fok1 polymorphism with breast cancer ( ff + Ff vs. FF ). (TIF) [file pone.0096125.s002.tif]

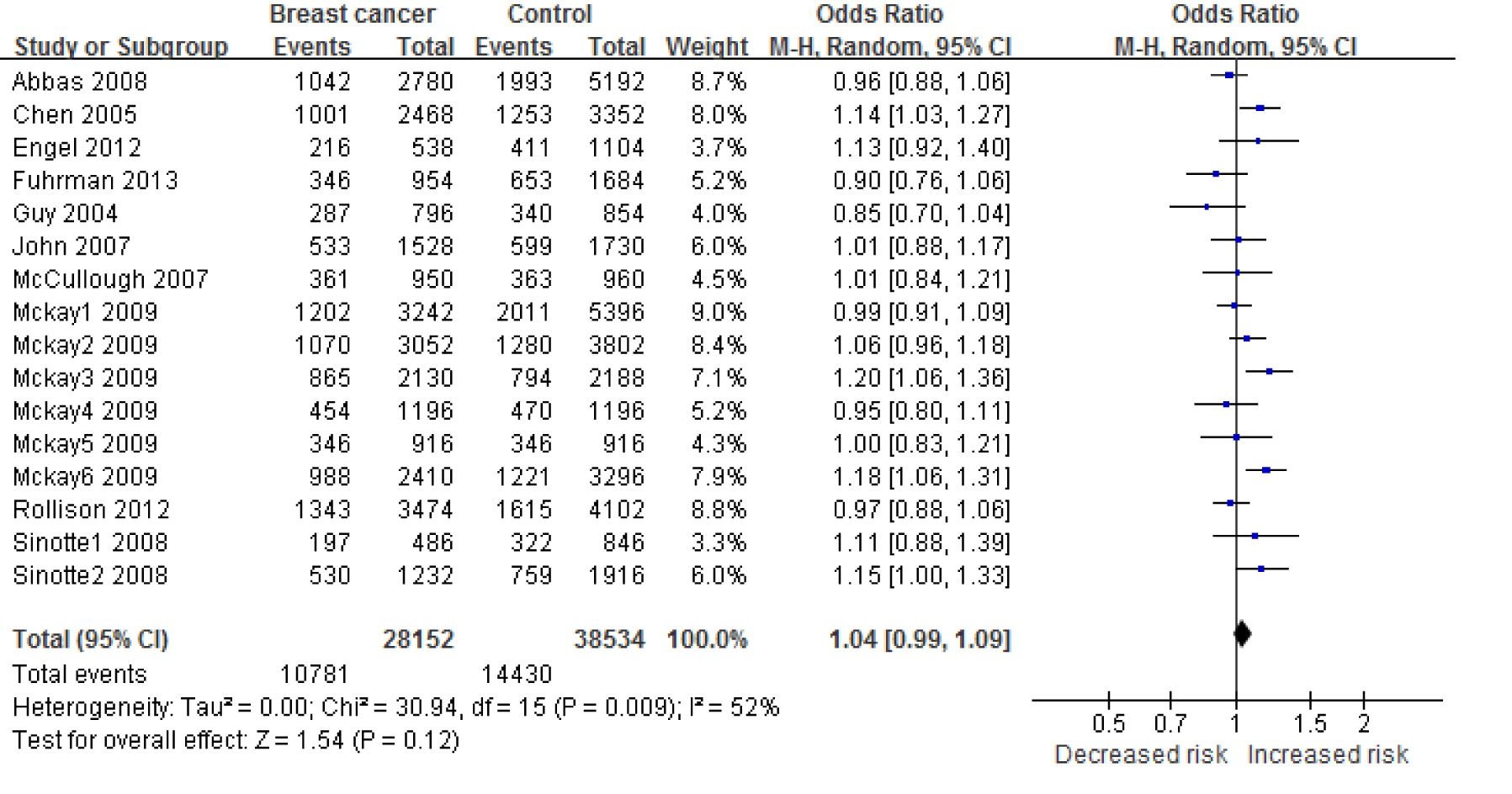

Supplement: Figure S3 — Forest plots of association of Fok1 polymorphism with breast cancer ( f vs. F ). (TIF) [file pone.0096125.s003.tif]
