# Supplementary material for: Association between Vitamin D Receptor Gene Polymorphisms and Breast Cancer Risk: A Meta-Analysis of 39 Studies
Source: PLoS One. 2014 Apr 25;9(4):e96125. doi: 10.1371/journal.pone.0096125 (PMC4000223; doi:10.1371/journal.pone.0096125)
Supplement: Table S1 — Characteristics of studies included in this meta-analysis between the Fok1 polymorphism in the vitamin D receptor gene and breast cancer. (DOCX) [file pone.0096125.s004.docx]

**Table S1** The characteristics of Fok1 polymorphism genotype distribution for breast cancer risk in studies included in this meta-analysis

| Athours[ref.] | Year | Country | Racial | Breast cancer  cancer | | |  |  | Control |  | p_-HWE_ |
| --- | --- | --- | --- | --- | --- | --- | --- | --- | --- | --- | --- |
|  |  |  | descent | n | FF/Ff/ff | F/f (%)  F/f(%) |  | n | FF/Ff/ff | F/f (%) |  |
| Curran et al.[9] | 1999 | Australia | European | 135 | 40/74/21 | 57.0/43.0 |  | 104 | 32/55/17 | 57.2/42.8 | 0.41 |
| Ingel et al. [10] | 2000 | America | European | 143 | 53/65/25 | 59.8/40.2 |  | 300 | 107/147/46 | 60.2/39.8 | 0.70 |
| Guy et al. [22] | 2004 | UK | European | 398 | 163/183/52 | 60.2/39.8 |  | 427 | 159/196/72 | 63.9/36.1 | 0.95 |
| Chen et al. [11] | 2005 | Tukey | European | 1234 | 440/587/207 | 59.4/40.6 |  | 1676 | 661/777/238 | 62.6/37.4 | 0.69 |
| John et al. [12] | 2007 | America | Mixed | 764 | 332/331/101 | 65.1/34.9 |  | 865 | 375/381/109 | 65.4/34.6 | 0.43 |
| McCullough et al. [13] | 2007 | America | European | 475 | 191/207/77 | 62.0/38.0 |  | 480 | 185/227/68 | 62.2/37.8 | 0.90 |
| Abbas et al. [14] | 2008 | Germany | European | 1390 | 566/606/218 | 62.5/37.5 |  | 2596 | 998/1203/395 | 61.6/38.4 | 0.30 |
| Sinotte et al. [15]^a^ | 2008 | Canada | European | 243 | 92/105/46 | 59.5/40.5 |  | 423 | 164/196/63 | 61.9/38.1 | 0.72 |
| Sinotte et al. [15]^b^ | 2008 | Canada | European | 616 | 198/306/112 | 57.0/43.0 |  | 958 | 353/451/154 | 60.4/39.6 | 0.62 |
| Mckay et al. [16]^a^ | 2009 | Unknown | European | 1621 | 643/754/224 | 62.9/37.1 |  | 2698 | 1070/1245/383 | 62.7/37.3 | 0.50 |
| Mckay et al. [16]^b^ | 2009 | America | European | 1526 | 657/668/201 | 64.9/35.1 |  | 1901 | 844/834/223 | 66.3/33.7 | 0.44 |
| Mckay et al. [16]^c^ | 2009 | America | European | 1065 | 380/505/180 | 59.4/40.6 |  | 1094 | 441/512/141 | 63.7/36.3 | 0.69 |
| Mckay et al. [16]^d^ | 2009 | America | European | 598 | 225/292/81 | 62.0/38.0 |  | 598 | 219/288/91 | 60.7/39.3 | 0.82 |
| Mckay et al. [16]^e^ | 2009 | America | European | 458 | 185/200/73 | 62.2/37.8 |  | 458 | 178/214/66 | 62.2/37.8 | 0.90 |
| Mckay et al. [16]^f^ | 2009 | America | European | 1205 | 422/578/205 | 59.0/61.0 |  | 1648 | 655/765/228 | 63.0/7.0 | 0.85 |
| Li et al. [23] | 2010 | China | Asian | 81 | 16/48/17 | 49.4/50.6 |  | 78 | 26/44/8 | 61.5/38.5 | 0.09 |
| Anderson et al. [17] | 2011 | Canada | European | 1546 | 602/747/197 | 63.1/36.1 |  | 1627 | 606/741/280 | 60.0/40.0 | 0.04 |
| Engel et al. [18] | 2012 | America | European | 269 | 93/136/40 | 59.9/40.1 |  | 552 | 218/257/77 | 62.8/37.2 | 0.93 |
| Rollison et al. [19] | 2012 | America | European | 1737 | 662/807/268 | 61.3/38.7 |  | 2051 | 752/983/316 | 60.6/39.4 | 0.86 |
| Fuhrman et al. [21] | 2013 | America | European | 477 | 201/206/70 | 63.7/36.3 |  | 842 | 323/385/134 | 61.2/38.8 | 0.28 |
| Mirash et al [24] | 2013 | Amierica | Mixed | 232 | 95/110/27 | 64.7/35.3 |  | 349 | 148/144/57 | 63.0/37.0 | 0.03 |
| Shahabazi et al. [20] | 2013 | Iran | Asian | 140 | 74/55/11 | 72.5/27.5 |  | 156 | 84/57/15 | 72.1/27.9 | 0.25 |

p_-HWE:_ p for Hardy Weinberg Equilibrium
