# Supplementary material for: Association between Vitamin D Receptor Gene Polymorphisms and Breast Cancer Risk: A Meta-Analysis of 39 Studies
Source: PLoS One. 2014 Apr 25;9(4):e96125. doi: 10.1371/journal.pone.0096125 (PMC4000223; doi:10.1371/journal.pone.0096125)
Supplement: Table S2 — Characteristics of studies included in this meta-analysis between the poly-A polymorphism in the vitamin D receptor gene and breast cancer. (DOCX) [file pone.0096125.s005.docx]

**Table S2** The characteristics of poly A polymorphism genotype distribution for breast cancer risk in studies included in this meta-analysis

| Athours[ref.] | Year | Country | Racial  descent | Breast cancer  cancer | | |  |  | Control |  | p_-HWE_ |
| --- | --- | --- | --- | --- | --- | --- | --- | --- | --- | --- | --- |
|  |  |  |  | n | LL/SL/SS | L/S (%) |  | n | LL/SL/SS | L/S (%) |  |
| Ingles et al. [10] | 2000 | American | European | 143 | 62/65/16 | 66.1/33.9 |  | 300 | 171/113/16 | 75.8/24.2 | 0.63 |
| Guy et al. [22] | 2004 | UK | European | 398 | 169/179/50 | 64.9/35.1 |  | 427 | 141/215/71 | 58.2/41.8 | 0.47 |
| Trabert et al. [25]^a^ | 2007 | America | European | 1139 | 440/532/167 | 62.0/38.0 |  | 905 | 354/402/149 | 61.3/38.7 | 0.06 |
| Trabert et al. [25]^b^ | 2007 | America | European | 441 | 225/181/35 | 71.5/28.5 |  | 417 | 221/169/27 | 73.3/26.7 | 0.48 |
| Wedren et al [27] | 2007 | Sweden | European | 1496 | 422/698/376 | 48.5/51.5 |  | 1340 | 388/651/301 | 46.8/33.2 | 0.37 |
| Chakraborty et al.[26] | 2009 | India | Asian | 160 | 3179/50/79 | 35.0/65.0 |  | 140 | 11/59/70 | 28.9/71.1 | 0.77 |
| Rollison et al. [19] | 2011 | America | European | 1716 | 699/773/244 | 63.3/36.7 |  | 2037 | 888/875/274 | 65.1/34.9 | 0.01 |

p_-HWE:_ p for Hardy Weinberg Equilibrium
