# Supplementary material for: Association between Vitamin D Receptor Gene Polymorphisms and Breast Cancer Risk: A Meta-Analysis of 39 Studies
Source: PLoS One. 2014 Apr 25;9(4):e96125. doi: 10.1371/journal.pone.0096125 (PMC4000223; doi:10.1371/journal.pone.0096125)
Supplement: Table S3 — Characteristics of studies included in this meta-analysis between the Bsm1 polymorphism in the vitamin D receptor gene and breast cancer. (DOCX) [file pone.0096125.s006.docx]

**Table S3** The characteristics of Bsm1 polymorphism genotype distribution for breast cancer risk in studies included in this meta-analysis

| Athours[ref.] | Year | Country | Racial  descent | Breast cancer  cancer | | |  |  | Control |  | p-_HWE_ |
| --- | --- | --- | --- | --- | --- | --- | --- | --- | --- | --- | --- |
|  |  |  |  | n | BB/Bb/bb | B/b (%) |  | n | BB/Bb/bb | B/b (%) |  |
| Ruggiero et al. [28] | 1998 | Italy | European | 88 | 12/51/25 | 42.6/57.4 |  | 167 | 20/121/26 | 48.2/51.8 | <0.0001 |
| Ingles et al. [10] | 2000 | America | European | 143 | 14/68/61 | 33.6/66.4 |  | 300 | 19/112/169 | 25.0/75.0 | 0.94 |
| Hou et al. [29] | 2002 | Taiwan | Asian | 34 | 1/6/27 | 11.8/88.2 |  | 169 | 0/16/153 | 4.7/95.3 | 0.52 |
| Buyru et al.[30] | 2003 | Turkey | European | 78 | 15/45/18 | 48.1/51.9 |  | 27 | 5/17/5 | 50.0/50.0 | 0.18 |
| Guy et al. [22] | 2004 | UK | European | 398 | 52/173/173 | 42.3/57.7 |  | 427 | 73/215/139 | 34.8/65.2 | 0.40 |
| Hefler et al. [31] | 2004 | Germany | European | 290 | 65/92/133 | 38.3/61.7 |  | 1699 | 255/860/584 | 40.3/59.7 | 0.03 |
| Chen et al. [11] | 2005 | Turkey | European | 1180 | 163/586/431 | 38.6/61.4 |  | 1547 | 245/737/565 | 39.7/60.3 | 0.86 |
| Lowe et al. [32] | 2005 | UK | European | 179 | 25/70/84 | 33.5/66.5 |  | 179 | 28/99/52 | 43.3/56.7 | 0.09 |
| Vandevord et al. [33] | 2006 | America | Mixed | 220 | 36/88/96 | 36.4/63.6 |  | 192 | 40/66/86 | 38.0/62.0 | <0.0001 |
| McCullough et al. [13] | 2007 | America | European | 472 | 84/237/151 | 42.9/57.1 |  | 460 | 74/216/170 | 39.6/60.4 | 0.70 |
| Trabert et al. [25]^a^ | 2007 | America | European | 1136 | 278/432/426 | 43.5/56.5 |  | 965 | 258/371/336 | 46.0/54.0 | <0.0001 |
| Trabert et al. [25]^b^ | 2007 | America | European | 485 | 67/174/244 | 31.8/63.2 |  | 446 | 50/168/228 | 30.0/70.0 | 0.03 |
| Sinotte et al. [15]^a^ | 2008 | Canada | European | 237 | 36/109/92 | 38.2/61.8 |  | 438 | 87/192/159 | 41.8/58.2 | 0.04 |
| Sinotte et al. [15]^b^ | 2008 | Canada | European | 617 | 80/300/237 | 37.3/62.7 |  | 956 | 140/461/355 | 38.8/61.2 | 0.62 |
| Mckay et al. [16]^a^ | 2009 | Unknown | European | 1596 | 256/767/573 | 40.1/59.9 |  | 2620 | 450/1219/951 | 40.4/59.6 | 0.08 |
| Mckay et al. [16]^b^ | 2009 | America | Unknown | 1536 | 115/518/903 | 24.3/75.7 |  | 1881 | 158/672/1051 | 26.3/73.7 | <0.0001 |
| Mckay et al. [16]^c^ | 2009 | America | European | 1065 | 192/468/405 | 40.0/60.0 |  | 1097 | 157/533/407 | 38.6/61.4 | 0.41 |
| Mckay et al. [16]^d^ | 2009 | America | European | 604 | 100/303/201 | 41.6/58.4 |  | 604 | 106/298/200 | 42.2/57.8 | 0.78 |
| Mckay et al. [16]^e^ | 2009 | America | European | 432 | 78/212/142 | 42.6/57.4 |  | 432 | 70/200/162 | 39.4/60.6 | 0.53 |
| Mckay et al. [16]^f^ | 2009 | America | European | 1122 | 160/555/407 | 39.0/61.0 |  | 1515 | 242/723/550 | 39.8/60.2 | 0.86 |
| Anderson et al.[17] | 2011 | Canada | European | 1553 | 269/746/538 | 41.3/58.7 |  | 1629 | 288/749/592 | 40.7/59.3 | 0.06 |
| Rollison et al. [19] | 2012 | America | European | 1740 | 247/809/684 | 37.4/62.6 |  | 2047 | 278/905/864 | 35.7/64.3 | 0.10 |
| Fuhrman et al. [21] | 2013 | America | European | 466 | 67/214/185 | 37.3/62.7 |  | 838 | 144/404/290 | 41.3/58.7 | 0.87 |
| Mirash et al. [24] | 2013 | America | Mixed | 349 | 26/141/182 | 27.7/72.3 |  | 232 | 19/90/123 | 27.6/72.4 | 0.66 |
| Shahbazi et al. [20] | 2013 | Iran | Asian | 140 | 16/73/51 | 37.5/62.5 |  | 156 | 36/72/48 | 46.2/53.8 | 0.37 |

p_-HWE:_ p for Hardy Weinberg Equilibrium
