# Supplementary material for: Association between Vitamin D Receptor Gene Polymorphisms and Breast Cancer Risk: A Meta-Analysis of 39 Studies
Source: PLoS One. 2014 Apr 25;9(4):e96125. doi: 10.1371/journal.pone.0096125 (PMC4000223; doi:10.1371/journal.pone.0096125)
Supplement: Table S4 — Characteristics of studies included in this meta-analysis between the Taq1 polymorphism in the vitamin D receptor gene and breast cancer. (DOCX) [file pone.0096125.s007.docx]

**Table S4** The characteristics of Taq1 polymorphism genotype distribution for breast cancer risk in studies included in this meta-analysis

| Athours[ref.] | Year | Country | Racial  descent | Breast cancer  cancer | | |  |  | Control |  | p_-HWE_ |
| --- | --- | --- | --- | --- | --- | --- | --- | --- | --- | --- | --- |
|  |  |  |  | n | TT/Tt/tt | T/t (%) |  | n | TT/Tt/tt | T/t (%) |  |
| Curran et al. [9] | 1999 | Australia | European | 135 | 53/67/15 | 64.1/35.9 |  | 110 | 30/62/18 | 55.5/44.5 | 0.14 |
| Dunning et al. [34]^a^ | 1999 | UK | European | 211 | 85/99/27 | 63.7/36.3 |  | 268 | 105/121/42 | 61.8/38.2 | 0.47 |
| Dunning et al. [34]^b^ | 1999 | UK | European | 740 | 260/354/126 | 59.1/40.9 |  | 359 | 128/172/59 | 59.6/40.4 | 0.92 |
| Lundin et al. [35] | 1999 | Sweden | European | 130 | 41/66/23 | 56.9/43.1 |  | 111 | 34/59/18 | 57.2/42.8 | 0.37 |
| Hou et al. [29] | 1999 | Taiwan | Asian | 34 | 29/5/0 | 92.6/7.4 |  | 167 | 154/13/0 | 91.1/3.9 | 0.60 |
| Cui et al. [38] | 2001 | China | Asian | 86 | 72/12/2 | 90.7/9.3 |  | 134 | 129/5/0 | 1.9/90.1 | 0.83 |
| Buyru et al. [30] | 2003 | Turkey | European | 78 | 26/44/8 | 61.5/38.5 |  | 27 | 4/20/3 | 51.9/48.1 | 0.01 |
| Sillanpaa et al. [36] | 2004 | Finnish | European | 472 | 212/226/34 | 68.9/31.1 |  | 479 | 218/214/47 | 67.8/32.2 | 0.60 |
| John et al. [12] | 2007 | America | Mixed | 762 | 350/319/93 | 66.9/33.1 |  | 863 | 376/401/86 | 66.8/33.2 | 0.16 |
| McCullough et al. [13] | 2007 | America | European | 494 | 85/251/158 | 42.6/57.4 |  | 492 | 81/231/180 | 39.9/60.1 | 0.64 |
| Abbas et al. [14] | 2008 | Germany | European | 1403 | 497/667/239 | 59.2/40.8 |  | 2609 | 980/1218/411 | 60.9/39.1 | 0.31 |
| Chakraborty et al. [26] | 2009 | India | Asian | 160 | 86/67/7 | 74.7/25.3 |  | 140 | 80/54/6 | 76.4/23.6 | 0.40 |
| Anderson et al. [17] | 2011 | Canada | European | 1556 | 552/744/260 | 59.4/40.6 |  | 1631 | 594/763/274 | 59.8/40.2 | 0.28 |
| Liu et al. [37] | 2011 | China | Asian | 80 | 29/39/12 | 60.6/39.4 |  | 80 | 31/37/12 | 61.9/38.1 | 0.09 |
| Engel et al. [18] | 2012 | America | European | 269 | 98/117/54 | 58.2/41.8 |  | 546 | 211/226/109 | 59.3/40.7 | 0.001 |
| Mirash et al.[24] | 2013 | America | Ruropean | 349 | 223/106/20 | 79.1/20.9 |  | 232 | 141/66/25 | 89.2/10.8 | 0.0002 |

p_-HWE:_ p for Hardy Weinberg Equilibrium
