# Supplementary material for: Association between Vitamin D Receptor Gene Polymorphisms and Breast Cancer Risk: A Meta-Analysis of 39 Studies
Source: PLoS One. 2014 Apr 25;9(4):e96125. doi: 10.1371/journal.pone.0096125 (PMC4000223; doi:10.1371/journal.pone.0096125)
Supplement: Table S5 — Characteristics of studies included in this meta-analysis between the Apa1 polymorphism in the vitamin D receptor gene and breast cancer. (DOCX) [file pone.0096125.s008.docx]

**Table S5** The characteristics of Apa1 polymorphism genotype distribution for breast cancer risk in studies included in this meta-analysis

| Athours[ref.] | Year | Country | Racial  descent | Breast cancer  cancer | | |  |  | Control |  | p_-HWE_ |
| --- | --- | --- | --- | --- | --- | --- | --- | --- | --- | --- | --- |
|  |  |  |  | n | AA/Aa/aa | A/a (%) |  | n | AA/Aa/aa | A/a (%)  A/a (%) |  |
| Curran et al. [9] | 1999 | Australia | European | 138 | 33/70/35 | 49.3/50.7 |  | 110 | 37/56/17 | 59.1/40.9 | 0.58 |
| Cui et al.[38] | 2001 | China | Asian | 86 | 12/38/36 | 36.0/64.0 |  | 134 | 21/53/60 | 35.4/64.6 | 0.12 |
| Hou et al. [29] | 2002 | Taiwan | Asian | 34 | 7/10/17 | 35.3/64.7 |  | 134 | 14/71/49 | 36.9/63.1 | 0.11 |
| Sillanpaa et al. [36] | 2004 | Finnish | European | 478 | 135/251/92 | 54.5/45.5 |  | 477 | 161/227/89 | 57.5/42.5 | 0.57 |
| McCullough et al. [13] | 2007 | America | European | 488 | 147/248/93 | 55.5/44.5 |  | 477 | 129/248/100 | 53.0/47.0 | 0.34 |
| Chakraborty et al. [26] | 2009 | India | Asian | 160 | 79/72/9 | 71.9/28.1 |  | 140 | 75/60/5 | 75.0/25.0 | 0.09 |
| Anderson et al. [17] | 2011 | Canada | European | 1544 | 438/766/340 | 53.2/46.8 |  | 1622 | 455/803/364 | 52.8/47.2 | 0.79 |
| Engel et al. [18] | 2012 | America | European | 268 | 88/120/60 | 55.2/44.8 |  | 552 | 160/251/141 | 51.7/48.3 | 0.04 |
| Dalessandri et al. [36] | 2012 | Canada | European | 164 | 47/84/33 | 54.3/45.7 |  | 174 | 67/73/34 | 59.5/40.5 | 0.09 |
| Huang et al.[40] | 2012 | China | Asian | 146 | 90/34/22 | 73.3/26.7 |  | 320 | 148/132/40 | 66.9/33.1 | 0.22 |
| Mirash et al.[24] | 2013 | America | European | 232 | 82/115/35 | 60.2/39.9 |  | 349/ | 110/185/54 | 58.0/42.0 | 0.09 |

p_-HWE:_ p for Hardy Weinberg Equilibrium
